# Effect of Methylfolate, Pyridoxal-5′-Phosphate, and Methylcobalamin (Soloways^TM^) Supplementation on Homocysteine and Low-Density Lipoprotein Cholesterol Levels in Patients with Methylenetetrahydrofolate Reductase, Methionine Synthase, and Methionine Synthase Reductase Polymorphisms: A Randomized Controlled Trial

**DOI:** 10.3390/nu16111550

**Published:** 2024-05-21

**Authors:** Evgeny Pokushalov, Andrey Ponomarenko, Sevda Bayramova, Claire Garcia, Inessa Pak, Evgenya Shrainer, Marina Ermolaeva, Dmitry Kudlay, Michael Johnson, Richard Miller

**Affiliations:** 1 Center for New Medical Technologies, 630090 Novosibirsk, Russia; ponomarenko_av@cnmt.ru (A.P.); bayramova_sa@cnmt.ru (S.B.); inesspak@yandex.ru (I.P.); shrayner_ev@cnmt.ru (E.S.); marermo@bk.ru (M.E.); 2 Scientific Research Laboratory, Triangel Scientific, San Francisco, CA 94101, USA; info@triangelcompany.com (C.G.);; 3 Institute of Pharmacy, I.M. Sechenov First Moscow State Medical University (Sechenov University), 119435 Moscow, Russia; d624254@gmail.com

**Keywords:** MTHFR, MTR, MTRR polymorphisms, methylfolate, pyridoxal-5′-phosphate, methylcobalamin, homocysteine, LDL-C, triglycerides, cardiovascular health, personalized medicine

## Abstract

Exploring the link between genetic polymorphisms in folate metabolism genes (MTHFR, MTR, and MTRR) and cardiovascular disease (CVD), this study evaluates the effect of B vitamin supplements (methylfolate, pyridoxal-5′-phosphate, and methylcobalamin) on homocysteine and lipid levels, potentially guiding personalized CVD risk management. In a randomized, double-blind, placebo-controlled trial, 54 patients aged 40–75 with elevated homocysteine and moderate LDL-C levels were divided based on MTHFR, MTR, and MTRR genetic polymorphisms. Over six months, they received either a combination of methylfolate, P5P, and methylcobalamin, or a placebo. At the 6 months follow-up, the treatment group demonstrated a significant reduction in homocysteine levels by 30.0% (95% CI: −39.7% to −20.3%) and LDL-C by 7.5% (95% CI: −10.3% to −4.7%), compared to the placebo (*p* < 0.01 for all). In the subgroup analysis, Homozygous Minor Allele Carriers showed a more significant reduction in homocysteine levels (48.3%, 95% CI: −62.3% to −34.3%, *p* < 0.01) compared to mixed allele carriers (18.6%, 95% CI: −25.6% to −11.6%, *p* < 0.01), with a notable intergroup difference (29.7%, 95% CI: −50.7% to −8.7%, *p* < 0.01). LDL-C levels decreased by 11.8% in homozygous carriers (95% CI: −15.8% to −7.8%, *p* < 0.01) and 4.8% in mixed allele carriers (95% CI: −6.8% to −2.8%, *p* < 0.01), with a significant between-group difference (7.0%, 95% CI: −13.0% to −1.0%, *p* < 0.01). Methylfolate, P5P, and methylcobalamin supplementation tailored to genetic profiles effectively reduced homocysteine and LDL-C levels in patients with specific MTHFR, MTR, and MTRR polymorphisms, particularly with homozygous minor allele polymorphisms.

## 1. Introduction

Cardiovascular diseases (CVDs) are a leading cause of mortality globally. Key risk factors contributing to this burden include elevated homocysteine and low-density lipoprotein cholesterol (LDL-C) levels [1]. Specifically, elevated homocysteine levels are independently associated with an increased risk of CVDs, including coronary artery disease, stroke, and peripheral vascular disease [2].

The metabolism of homocysteine is closely linked to the availability of B vitamins such as folate, vitamin B6, and vitamin B12 [3]. Variants in genes like methylenetetrahydrofolate reductase (MTHFR), methionine synthase (MTR), and methionine synthase reductase (MTRR) play significant roles in folate metabolism and, thus, influence homocysteine levels [4]. The MTHFR gene, for instance, is crucial for converting 5,10-methylenetetrahydrofolate to 5-methyltetrahydrofolate, a key step in homocysteine regulation. Similarly, the MTR and MTRR genes are involved in the folate cycle, impacting homocysteine synthesis and metabolism. Disruptions in these pathways, often due to gene polymorphisms, can lead to elevated homocysteine levels [5].

B vitamin supplementation, notably with methylfolate, pyridoxal-5′-phosphate (P5P), and methylcobalamin, has been shown to significantly reduce homocysteine levels, operating through distinct biochemical pathways [6]. Methylfolate, a bioactive form of folate, directly contributes to the remethylation of homocysteine to methionine. This process is particularly crucial for patients with MTHFR polymorphisms, as these genetic variations can impair the conversion of folate to its active form, leading to elevated homocysteine levels. By providing the body with readily available methylfolate, this metabolic block is bypassed, facilitating the efficient conversion of homocysteine to methionine [6].

Pyridoxal-5′-phosphate (P5P), the active form of vitamin B6, plays a pivotal role in the trans-sulfuration pathway, where homocysteine is converted into cysteine. This pathway becomes particularly important in conditions where the primary remethylation pathway is less efficient, such as in patients with MTHFR polymorphisms [7].

Methylcobalamin, an active form of vitamin B12, is another essential cofactor in the remethylation of homocysteine to methionine. It works synergistically with methylfolate in this pathway [8].

Together, these supplements address the key enzymatic steps in homocysteine metabolism, providing a comprehensive approach to lowering homocysteine levels, particularly in those with genetic predispositions that disrupt normal folate and homocysteine metabolism [8].

This study investigated the impact of methylfolate, pyridoxal-5′-phosphate, and methylcobalamin on homocysteine levels in patients with a low to medium cardiac risk and MTHFR, MTR, and MTRR gene polymorphisms. As a randomized, double-blind, placebo-controlled trial, it focused on the efficacy of these vitamins in reducing homocysteine, a key CVD risk factor, while also monitoring LDL-C levels. The results could provide valuable insights for personalized cardiovascular disease prevention and management, highlighting the role of genetics in nutritional therapy.

## 2. Materials and Methods

This was a randomized, double-blind, parallel-group clinical trial that compared treatment with methylfolate, P5P, and methylcobalamin to a placebo. The study protocol was approved by the local Ethics Committee and was conducted in compliance with the protocol, standard institutional operating procedures, and the Declaration of Helsinki. All patients enrolled in the study provided written informed consent. The study was registered with ClinicalTrials.gov (NCT06163443).

### 2.1. Patient Population and Design

Patients were eligible based on the following criteria:

Inclusion criteria:

- Age between 40 and 75;
- Homocysteine levels greater than 15 µmol/L and LDL-C level between 70 and 190 mg/dL, confirmed in at least two sequential checks conducted within the last six months prior to signing the consent form;
- Presence of at least one minor allele in any of the following genetic polymorphisms: rs1801133 (MTHFR C677T), rs1801131 (MTHFR A1298C), rs1805087 (MTR A2756G), and rs1801394 (MTRR A66G) [4].

Exclusion criteria:

- Personal history of cardiovascular disease or high risk (≥20%);
- Triglycerides (TG) ≥ 400 mg/dL;
- Obesity (Body Mass Index > 30 kg/m^2^);
- Assumption of lipid-lowering drugs or supplements affecting lipid metabolism within the last three months;
- Use of medications or supplements known to affect homocysteine levels, such as B-vitamins and certain antihypertensives, within the last three months;
- Diabetes mellitus;
- Known severe or uncontrolled thyroid, liver, renal, or muscle diseases.

**Figure 1:**
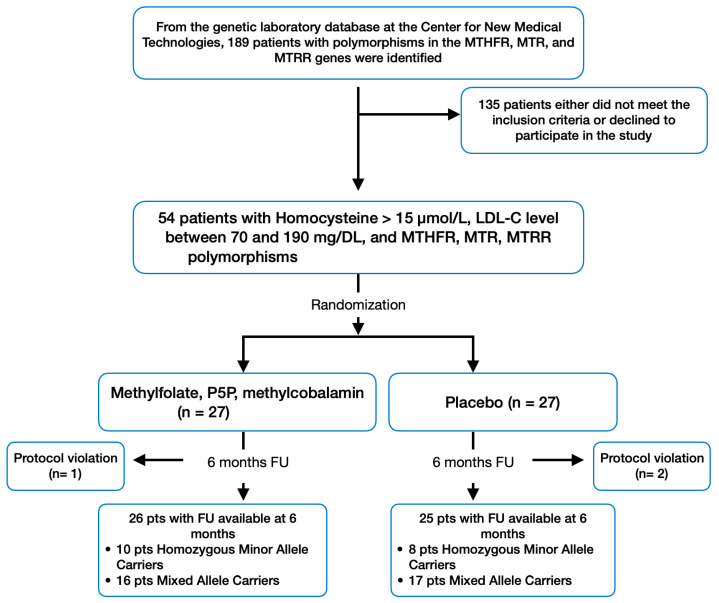
Study design and patient flow.

In the study, patients with polymorphisms in the MTHFR, MTR, and MTRR genes were identified from the database of the Center for New Medical Technologies’ genetic laboratory. Following their consent to participate and if they met the inclusion and exclusion criteria, these patients were enrolled in the research. A total of 54 patients were included in the study. Subsequently, they were randomized to methylfolate, P5P, and methylcobalamin (*n* = 27), or placebo (*n* = 27) groups using a computer-generated random sequence (Figure 1). The treatment allocations were blinded to both the researchers and the patients throughout the study. Participants in each group took 2 capsules/day. Methylfolate, P5P, and methylcobalamin and placebo capsules were identical in appearance, matched for color coating, shape, and size. The active treatment, provided by S.Lab (Soloways^TM^, Novosibirsk, Russia), LLC, contained L-methylfolate (1 mg), P5P (pyridoxal-5-phosphate, 50 mg), and Methylcobalamin (500 mcg) in each capsule. The duration of the study was 180 days. All patients were instructed to maintain their usual diet, lifestyle, and medication. The consumption of supplements and placebos during the study was monitored by asking people to return the medication containers and through brief daily cell phone reminders for participants to take the supplements.

Participants underwent fasting measurements of their complete lipid profile, including low-density lipoprotein cholesterol (LDL-C), calculated using the Friedewald equation; a complete metabolic panel; homocysteine levels; and high-sensitivity *C*-reactive protein (hsCRP), on day 0, day 90, and day 180 of the study.

In the study, participants were categorized into two distinct genetic groups for our predefined sub-analysis—the Homozygous Minor Allele Carriers subgroup includes patients who have two copies of the minor allele for at least one of the specific genes under study; the mixed allele carriers subgroup comprises all other patients. This category is not limited to heterozygous genotypes for all genes but includes any genetic configuration that is not homozygous minor. These categorizations, as pre-specified in the study protocol, were instrumental in facilitating a detailed assessment of the therapy’s effectiveness in relation to these specific genetic profiles [9,10].

In this study, S.Lab (Soloways ^TM^), a pharmaceutical company, contributed solely by manufacturing the dietary supplements (methylfolate, P5P, and methylcobalamin) used in the research. S.Lab (Soloways ^TM^) did not participate in the design, execution, or financing of the experiment, beyond providing the required supplements. The entire study was independently conducted by a research team from the Center for New Medical Technologies and the Scientific Research Laboratory at Triangel Scientific. This arrangement ensured that the study’s outcomes were not influenced by commercial interests, maintaining the integrity and independence of our research.

### 2.2. Study Endpoints and Assessments

The primary endpoint was the percent change in homocysteine levels from baseline to 6 months of observation, comparing a combined treatment regimen of methylfolate, P5P, and methylcobalamin against a placebo among patients with MTHFR, MTR, and MTRR polymorphisms. Secondary endpoints included the percent changes in LDL-C, high-density lipoprotein cholesterol (HDL-C), total cholesterol, serum triglycerides, and hsCRP, measured from baseline to the 6-month mark between the combined treatment and placebo groups.

### 2.3. Sample Size Calculation and Statistical Power

For our randomized, placebo-controlled trial, the primary objective was to detect a 15% reduction in homocysteine levels in the active treatment group compared to the placebo group, assuming an average baseline homocysteine level of 18 μmol/L. We considered a standard deviation of 15% in homocysteine level changes across both groups. Our sample size calculation, using a two-sided significance level of 5% and aiming for a power of 90%, initially indicated that 21 participants would be needed in each group. To account for a potential dropout rate of 20%, we adjusted this number, rounding up to the nearest whole number. Consequently, we determined that enrolling 27 participants per group was necessary to maintain the desired power of the study. This adjustment resulted in a total sample size of 54 participants for the trial, ensuring robust statistical validity and the capacity to reliably detect the hypothesized effect of the intervention on homocysteine levels.

### 2.4. Statistical Analyses

The primary statistical analysis aimed to assess the impact of a combined treatment regimen of methylfolate, P5P, and methylcobalamin on homocysteine levels, comparing these effects against a placebo in patients with MTHFR, MTR, and MTRR polymorphisms. Analyses were conducted on an intention-to-treat basis, encompassing all participants who received at least one dose of the treatment or placebo and had at least one post-baseline efficacy evaluation. The percent changes in homocysteine and LDL-C levels from baseline to the 6-month follow-up were calculated, with comparisons between the treatment and placebo groups conducted using independent *t*-tests.

Secondary analyses evaluated changes in other lipid biomarkers and hsCRP levels. These analyses were carried out using independent *t*-tests for each biomarker. To compare the efficacy within subgroups stratified by genotype, a two-way ANOVA was performed with treatment and genotype as factors, without the need for post hoc tests with Bonferroni correction, as the changes in total cholesterol, HDL-C, triglycerides, and hsCRP did not reach statistical significance.

Interaction terms were included in the models to investigate potential genotype-related treatment effects. Subgroup analyses were specified for Homozygous Minor Allele Carriers and mixed allele carriers.

Statistical tests were two-tailed with a significance threshold set at *p* < 0.05. The study was powered to detect significant differences, accounting for the sample size and dropout rate. Continuous variables were presented as mean ± SD and categorical variables were presented as counts and percentages.

Statistical analyses were executed using appropriate statistical software SAS version 9.4 (SAS Institute, Cary, NC, USA), with data visualizations created to illustrate the distribution of changes and treatment effects, particularly focusing on the significant reductions in homocysteine and LDL-C levels in the treatment group and in specific genotypic subgroups.

This approach ensured a rigorous examination of treatment effects, with a particular emphasis on the influence of genetic polymorphisms on the metabolic responses to the combined treatment.

## 3. Results

A total of 54 participants initially enrolled in the study, with 51 successfully completing it, according to the protocol (Figure 1). High adherence to the prescribed regimen was observed and participants consistently maintained their dietary habits throughout the trial.

Upon analyzing the baseline characteristics, we found no statistically significant differences between the two groups regarding age, gender distribution, body mass index (BMI), or estimated 10-year ASCVD risk. This similarity indicates demographic and clinical homogeneity at the study’s outset.

Focusing on homocysteine levels, a primary interest in our study, we observed similar initial concentrations in both groups—18.5 ± 4.1 µmol/L in the treatment group and 19.1 ± 3.8 µmol/L in the placebo group (*p* = 0.81). We also assessed the lipid profiles, including total cholesterol, LDL-C, HDL-C, and triglycerides, alongside hsCRP levels. These assessments revealed no significant disparities between the two groups.

For the subgroup analysis, we categorized participants as either Homozygous Minor Allele Carriers (*n* = 18) or mixed allele carriers (*n* = 33). This stratification allowed us to explore potential baseline characteristic differences influenced by genetic variations. However, it is important to note that a detailed analysis of baseline characteristics in the Homozygous Carriers group is limited due to the small number of patients within each specific genetic variant. Specifically, the distribution is as follows: 1 patient with homozygous minor allele MTHFR C677T (A/A), 1 patient with homozygous minor allele MTHFR A1298C (G/G), 1 patient with homozygous minor allele MTR A2756G (G/G), 4 patients with homozygous minor allele MTRR A66G (G/G), and 11 patients with combinations of homozygous minor alleles. Consequently, while we observed trends such as higher baseline homocysteine levels in Homozygous Carriers (25.1 ± 4.1 µmol/L) compared to Mixed Carriers (17.3 ± 3.9 µmol/L, *p* < 0.01) and higher LDL-C levels in Homozygous Carriers (150.1 ± 29.3 mg/dL) versus Mixed Carriers (127.7 ± 26.8 mg/dL, *p* < 0.01), the limited sample size precludes definitive conclusions. Similarly, slightly lower HDL-C levels in Homozygous Carriers (47.6 ± 4.8 mg/dL) versus Mixed Carriers (52.2 ± 5.8 mg/dL, *p* = 0.05) must be interpreted with caution. Triglyceride and hsCRP levels did not show significant differences between these genetic subgroups.

Detailed results of these findings are presented in Table 1.

### 3.1. Primary Endpoint

**Figure 2:**
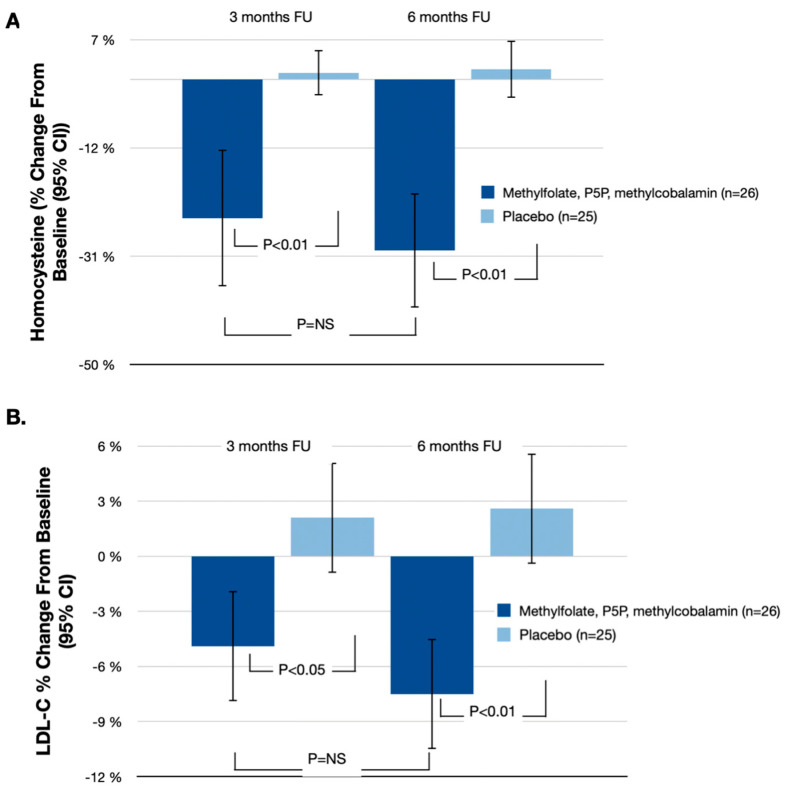
(**A**) Mean percent homocysteine change at 3 and 6 months follow-up; (**B**) Mean percent LDL-C change at 3 and 6 months follow-up. NS—Not Significant.

As depicted in Table 2 and Figure 2, there was a substantial percentage change in homocysteine levels from baseline to the 6 months follow-up. Patients in the treatment group receiving methylfolate, P5P, and methylcobalamin (*n* = 26) experienced a notable mean reduction in homocysteine levels of 30.0% (95% CI: −39.7% to −20.3%). Conversely, the placebo group (*n* = 25) had a marginal mean increase of 1.8% in homocysteine levels (95% CI: −4.8% to 6.8%). The difference in percentage change between the two groups was significant at 31.8% (95% CI: −46.5% to −15.5%; *p* < 0.01).

### 3.2. Other Secondary End Points

Table 2 and Figure 2 illustrate the percent change from baseline to 6 months follow-up in other lipid biomarkers (total cholesterol, LDL-C, HDL-C, and triglycerides) and hsCRP.

The treatment group showed a reduction in LDL-C by 7.5% (95% CI: −10.3% to −4.7%), which was statistically significant when compared with the 2.6% increase observed in the placebo group (95% CI: −1.6% to 5.6%). The difference in percentage change between the two groups was significant at 10.1% (95% CI: −15.9% to −3.1%; *p* < 0.01).

Total cholesterol levels decreased by 2.5% in the treatment group (95% CI: −4.8% to −0.3%), while the placebo group experienced a 2.1% increase (95% CI: −0.6% to 4.8%); however, this difference did not reach statistical significance (*p* = 0.08).

For HDL-C, the treatment group had a slight, non-significant increase of 1.6% (95% CI: −1.4% to 4.6%), compared to a 0.5% decrease in the placebo group (95% CI: −3.0% to 2.0%; *p* = 0.16). Similarly, serum triglyceride levels in the treatment group decreased by 3.7% (95% CI: −7.7% to 0.3%), opposed to a 2.8% increase in the placebo group (95% CI: −0.3% to 5.9%), but this difference was not statistically significant (*p* = 0.11).

The hsCRP levels showed a reduction of 5.3% in the treatment group (95% CI: −13.0% to 2.5%) compared to a reduction of 3.2% in the placebo group (95% CI: −8.3% to 1.9%), with no significant difference between the two groups (*p* = 0.23).

**Figure 3:**
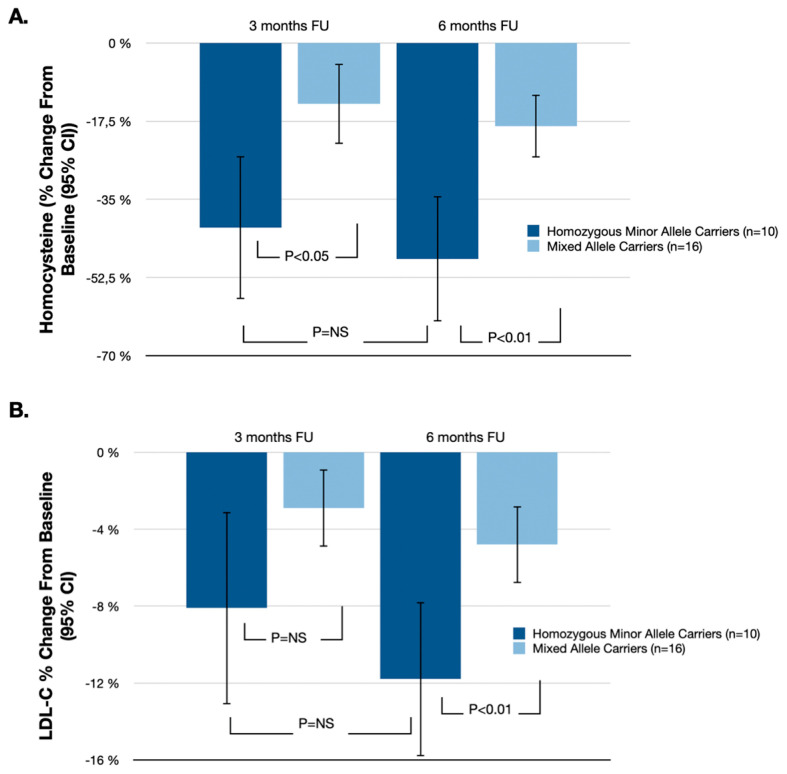
(**A**) Mean percent homocysteine change at 3 and 6 months follow-up; (**B**) mean percent LDL-C change at 3 and 6 months follow-up. NS—Not Significant.

In the subgroup analysis at 6 months follow-up (referenced as Table 3 and Figure 3), Homozygous Minor Allele Carriers showed a significant reduction in homocysteine levels of 48.3% (95% CI: −62.3%, −34.3%), as opposed to an 18.6% reduction in mixed allele carriers (95% CI: −25.6%, −11.6%). The intergroup differential was marked at 29.7% (95% CI: −50.7%, −8.7%; *p* < 0.01). LDL-C levels decreased by 11.8% (95% CI: −15.8%, −7.8%) in the homozygous subgroup and by 4.8% (95% CI: −6.8%, −2.8%) in the mixed allele carriers group, with a significant between-group difference of 7.0% (95% CI: −13.0%, −1.0%; *p* < 0.01). Changes in total cholesterol, HDL-C, triglycerides, and hsCRP were observed in both subgroups; however, none reached statistical significance.

## 4. Discussion

In our study, we not only observed significant changes in homocysteine levels, but also in LDL-C among participants. The augmented findings of this randomized, double-blind, parallel-group clinical trial can be summarized as follows: (1) patients with low to medium cardiac risk carrying MTHFR, MTR, and MTRR gene polymorphisms and receiving a combination of methylfolate, P5P, and methylcobalamin showed a notable reduction in homocysteine levels after 6 months of treatment; (2) patients with homozygous minor allele variants of these genes experienced a more substantial decrease in homocysteine levels, suggesting a significant benefit of personalized nutritional therapy; and (3) in addition to homocysteine reduction, there was a modest but statistically significant decrease in LDL-C levels in the treatment group, indicating the potential of B vitamin supplementation in improving lipid profiles and offering broader cardiovascular protective effects.

The considerable reduction in homocysteine levels observed in our research corroborates the existing literature on the efficacy of B vitamin supplementation, especially in those with genetic polymorphisms affecting folate metabolism. This aligns with the analysis of Wald et al., which emphasized the role of homocysteine reduction in the management and prevention of cardiovascular diseases [11]. Our study adds to this knowledge by showing the effectiveness of a combined B vitamin regimen in a genetically diverse patient group.

The distinct responses between homozygous minor allele carriers and mixed allele carriers are notable. Homozygous carriers exhibited a greater reduction in homocysteine levels, aligning with findings regarding the increased need for methylated folate in certain genetic variants, particularly in the MTHFR gene [12]. Frosst et al. also emphasized the role of MTHFR gene polymorphisms in folate metabolism and homocysteine levels, underlining the importance of genotype-specific approaches [13].

In addition to MTHFR, our study sheds light on the critical roles of MTR and MTRR enzymes in homocysteine metabolism. Methionine synthase (MTR) facilitates the remethylation of homocysteine to methionine, a process reliant on methylcobalamin. This pathway highlights the importance of MTR in maintaining normal homocysteine levels, with genetic variations potentially impacting its enzymatic efficiency [14]. Concurrently, methionine synthase reductase (MTRR) is essential for reactivating MTR by ensuring it remains in a functionally reduced state, critical for effective homocysteine management. The MTRR A66G polymorphism, for example, has been shown to significantly influence circulating homocysteine concentrations, underscoring its role in this metabolic pathway [15]. Furthermore, research indicates that the *N*-terminus of MTRR not only participates in electron transfer for MTR reactivation, but may also enhance MTR catalytic functions, adding another layer of regulatory complexity [16]. These pathways suggest a nuanced interplay between genetic polymorphisms and enzymatic activity that directly influences homocysteine levels.

The relationship between genetic polymorphisms in folate metabolism and cardiovascular disease (CVD) risk factors has been explored in studies like those by Jacques et al. and Chiuve et al. [17,18]. These investigations provide a framework for understanding the interaction between genetics, micronutrient metabolism, and cardiovascular health. Our study extends this framework by demonstrating the advantages of personalized B vitamin supplementation—particularly B12, B6, and folate—in significantly lowering homocysteine levels, especially in patients with specific genetic predispositions.

While our study successfully met its primary endpoint of reducing homocysteine levels, an intriguing secondary finding was the modest decrease in LDL-C levels observed in the treatment group. This outcome presents a novel aspect in the context of B vitamin supplementation research. Traditionally, investigations in this field have predominantly centered on the homocysteine-lowering effects of B vitamins [19]. The observed LDL-C reduction in our study, though modest, is significant and warrants further exploration.

The potential mechanisms underlying this LDL-C reduction remain speculative. One plausible explanation could be the role of B vitamins in modulating lipid metabolism. Studies have suggested that certain B vitamins can influence lipid profiles, although these effects are not as well characterized as their impact on homocysteine levels [20]. For instance, vitamin B12 and folate, in particular, have been implicated in lipid metabolism processes, potentially affecting LDL-C levels [21]. This association could be due to the interplay between these vitamins and lipid metabolism pathways, although the exact mechanisms are yet to be fully elucidated.

Furthermore, our findings did not reveal significant changes in other lipid parameters, such as high-density lipoprotein cholesterol, total cholesterol, triglycerides, or hsCRP levels. This observation aligns with the broader scientific consensus that B vitamin supplementation predominantly influences homocysteine levels, with a less pronounced effect on other cardiovascular risk markers [22]. However, the modest reductions in these markers observed in our study, although not reaching statistical significance, may have clinical relevance in a long-term context or in a larger cohort. The potential implications of these minor changes warrant further investigation, especially given the complex interplay between various lipid fractions and cardiovascular risk [23].

Our study employs a randomized, double-blind, placebo-controlled design for reliability and generalizability, reducing biases and enhancing data integrity. Genetic stratification based on MTHFR, MTR, and MTRR polymorphisms offers personalized insights into nutrient metabolism and disease risk. However, limitations include a small sample size, sufficient for homocysteine and LDL-C level analysis, but restrictive for broader genetic analysis, and a six-month duration, limiting insights into long-term effects and necessitating extended follow-up for the comprehensive evaluation of B vitamin supplementation impacts.

## 5. Conclusions

This study demonstrates that supplementation with methylfolate, pyridoxal-5′-phosphate, and methylcobalamin effectively reduces homocysteine levels, particularly in patients with MTHFR, MTR, and MTRR gene polymorphisms. While significant reductions in LDL-C levels were also observed, the impact on other lipid markers and hsCRP was not statistically significant. These findings underscore the importance of personalized nutritional interventions in cardiovascular disease prevention, especially in genetically susceptible populations.

## Figures and Tables

**Table 1:** Demographic and clinical characteristics of the participants at baseline.

	Methylfolate, P5P, Methylcobalamin (*n* = 26)	Placebo (*n* = 25)	*p*-Value	Homozygous Minor Allele Carriers (*n* = 18)	Mixed Allele Carriers (*n* = 33)	*p*-Value
Age, y	59.2 ± 6.2	57.5 ± 9.1	*p* = 0.45	57.3 ± 7.8	59.1 ± 8.9	*p* = 0.07
Women, %	57.7	52.0	*p* = 0.61	60	52	*p* = 0.21
Body mass index, kg/m^2^	27.5 ± 3.1	28.9 ± 3.3	*p* = 0.33	29.2 ± 3.2	27.5 ± 3.6	*p* = 0.09
10-y ASCVD risk, %	9.9	8.5	*p* = 0.75	10	8.5	*p* = 0.11
Homocysteine, μmol/L	18.5 ± 4.1	19.1 ± 3.8	*p* = 0.81	25.1 ± 4.1	17.3 ± 3.9	*p* < 0.01
Total cholesterol, mg/dL	180 ± 21	185 ± 31	*p* = 0.68	190 ± 29	180 ± 25	*p* = 0.08
LDL-C, mg/dL	125.4 ± 27.2	131.7 ± 29.1	*p* = 0.57	150.1 ± 29.3	127.7 ± 26.8	*p* < 0.01
HDL-C, mg/dL	52.4 ± 6.5	48.9 ± 4.1	*p* = 0.11	47.6 ± 4.8	52.2 ± 5.8	*p* = 0.05
Triglycerides, mg/dL	159 ± 32	148 ± 27	*p* = 0.42	165 ± 29	147 ± 25	*p* = 0.18
hsCRP, mg/L	1.8 ± 0.7	2.5 ± 1.1	*p* = 0.09	2.5 ± 0.9	2.0 ± 1.2	*p* = 0.23

**Table 2:** Primary and secondary endpoints at 180 days follow-up in main groups.

	Methylfolate, P5P, Methylcobalamin (*n* = 26) % Change from Baseline (95% CI)	Placebo (*n* = 25) % Change from Baseline (95% CI)	% Difference (95% CI)	*p*-Value
Homocysteine	−30.0% (−39.7%, −20.3%)	1.8% (−4.8%, 6.8%)	−31.8% (−46.5%, −15.5%)	*p* < 0.01
LDL-C	−7.5% (−10.3%, −4.7%)	2.6% (−1.6%, 5.6%)	−10.1% (−15.9%, −3.1%)	*p* < 0.01
Total cholesterol	−2.5% (−4.8%, −0.3%)	2.1% (−0.6%, 4.8%)	−4.6% (−9.6%, 0.3%)	*p* = 0.08
HDL-C	1.6% (−1.4%, 4.6%)	−0.5% (−3.0%, 2.0%)	2.1% (−3.4%, 7.6%)	*p* = 0.16
Triglycerides	−3.7% (−7.7%, 0.3%)	2.8% (−0.3%, 5.9%)	−6.5% (−13.6%, 0.6%)	*p* = 0.11
hsCRP	−5.3% (−13.0%, 2.5%)	−3.2% (−8.3%, 1.9%)	−2.1% (−14.9%, 10.8%)	*p* = 0.23

**Table 3:** Secondary endpoints at 6 months follow-up for homozygous and mixed minor allele carriers.

	Homozygous Minor Allele Carriers (*n* = 10) % Change from Baseline (95% CI)	Mixed Allele Carriers (*n* = 16) % Change from Baseline (95% CI)	% Difference (95% CI)	*p*-Value
Homocysteine	−48.3% (−62.3%, −34.3%)	−18.6% (−25.6%, −11.6%)	−29.7% (−50.7%, −8.7%)	*p* < 0.01
LDL-C	−11.8% (−15.8%, −7.8%)	−4.8% (−6.8%, −2.8%)	−7.0% (−13.0%, −1.0%)	*p* < 0.01
Total cholesterol	−3.2% (−5.6%, −0.8%)	−2.1% (−4.3%, 0.1%)	−1.1% (−5.7%, 3.5%)	*p* = 0.07
HDL-C	2.8% (−0.5%, 6.1%)	0.9% (−1.9%, 3.7%)	1.9% (−4.2%, 8.0%)	*p* = 0.12
Triglycerides	−5.1% (−10.9%, 0.7%)	−2.8% (−5.7%, 0.1%)	−2.3% (−11.0%, 6.4%)	*p* = 0.09
hsCRP	−3.9% (−11.6%, 3.8%)	−6.1% (−13.9%, 1.7%)	2.2% (−17.7%, 13.3%)	*p* = 0.22

## Data Availability

The data presented in this study are available on request from the corresponding author. The data are not publicly available as the participants did not consent to their data being shared publicly.
